# BOLSTERING SOCIAL CONNECTEDNESS DURING COVID-19: COMMUNICATION BETWEEN ESSENTIAL CONTACTS AND OLDER ADULTS

**DOI:** 10.1093/geroni/igac059.2186

**Published:** 2022-12-20

**Authors:** Joanna George, Sarah Hubner, Julie Blaskewicz Boron

**Affiliations:** Oklahoma State University College of Osteopathic Medicine, Tulsa, Oklahoma, United States; University of Nebraska Omaha, Omaha, Nebraska, United States; University of Nebraska, Omaha, Omaha, Nebraska, United States

## Abstract

Social isolation may increase morbidity and mortality, particularly for aging adults. Research suggests that COVID-19 has significantly disrupted social networks, exacerbating isolation and risk. However, the extent of disruption and its implications for older adults and their essential contacts (ECs) is unknown. ECs, those who provide support/engagement to older adults, play a significant role in networks and help shape communication patterns. Understanding the effects of COVID-19 on social connectedness between ECs and older adults is vital to promoting their well-being. The purpose of this study was to investigate ECs’ patterns of communication with adults aged 60+ during the COVID-19 pandemic , considering preferences, needs, and barriers. Self-identified ECs (N=546, Aged 19+, MAge=44.3±14.2) completed a Qualtrics survey via Amazon Mechanical Turk. Participants were ECs for community-dwelling (CDECs=57.3%) and institutionalized adults (IECs=42.7%). In addition to likert-style questions, the survey included free-response sections, examining communication quality, frequency, and method. Content analysis was conducted independently by two reviewers, using inductive coding. Qualitative results revealed distanced communication was widely utilized. Audiovisual/video communication was more frequently noted as desirable by IECs (32.8%) than CDECs (11.85%). Further, preference for audiovisual communication negatively correlated to expression of COVID-19-related barriers (IECs=12.5%, CDECs=7.7%, (r(546)= -.128, p=.01). Comparisons suggested that IECs reported absence of in-person contact with older adults more frequently than CDECs (recent contact=Never: 51% and 13.4% respectively). This may support the utility of video-communication to meaningfully supplement connectedness in the absence of in-person contact. The experiences described can reveal avenues for loneliness interventions and may guide future technology innovations.

